# The Change in Metabolic Syndrome Status and the Risk of Nonviral Liver Cirrhosis

**DOI:** 10.3390/biomedicines9121948

**Published:** 2021-12-20

**Authors:** Goh-Eun Chung, Young Chang, Yuri Cho, Eun-Ju Cho, Jeong-Ju Yoo, Sang-Hyun Park, Kyungdo Han, Dong-Wook Shin, Su-Jong Yu, Yoon-Jun Kim, Jung-Hwan Yoon

**Affiliations:** 1 Department of Internal Medicine, Healthcare Research Institute, Gangnam Healthcare Center, Seoul National University Hospital, Seoul 06236, Korea; gohwom@snu.ac.kr; 2 Department of Internal Medicine, Digestive Disease Center, Institute for Digestive Research, Soonchunhyang University College of Medicine, Seoul 04401, Korea; chyoung86@gmail.com; 3 Center for Liver and Pancreatobiliary Cancer, Research Institute and Hospital, National Cancer Center, Goyang 10408, Korea; presh_yuri@hanmail.net; 4 Department of Internal Medicine and Liver Research Institute, Seoul National University College of Medicine, Seoul 03080, Korea; creatioex@gmail.com (E.-J.C.); yoonjun@snu.ac.kr (Y.-J.K.); yoonjh@snu.ac.kr (J.-H.Y.); 5 Department of Gastroenterology and Hepatology, Soonchunhyang University Bucheon Hospital, Bucheon 14584, Korea; puby17@naver.com; 6 Department of Biostatistics, College of Medicine, The Soongsil University, Seoul 06591, Korea; ujk8774@naver.com (S.-H.P.); hkd917@naver.com (K.H.); 7 Samsung Medical Center, Department of Family Medicine, Sungkyunkwan University School of Medicine, Seoul 06351, Korea; 8 Department of Clinical Research Design & Evaluation, Samsung Advanced Institute for Health Science & Technology (SAIHST), Sungkyunkwan University, Seoul 06351, Korea; 9 Department of Digital Health, Samsung Advanced Institute for Health Science & Technology (SAIHST), Sungkyunkwan University, Seoul 06351, Korea

**Keywords:** metabolic syndrome, cirrhosis, incidence, nationwide

## Abstract

Background: Nonalcoholic fatty liver disease is considered to be the hepatic component of metabolic syndrome (MetS). However, the association between changes in MetS status and the risk of liver cirrhosis (LC) has not been investigated to date. This study assessed the association between changes in MetS and subsequent nonviral LC development. Methods: Data were obtained from the Korean National Health Insurance Service. Individuals who participated in health screenings from both 2009 to 2010 and 2011 to 2012 were included. The primary outcome was LC development according to the static and dynamic MetS status. Subjects were stratified into four groups according to the change in MetS status observed from the two-year interval screening (2009–2011). Cox regression analysis was used to examine the hazard ratios of LC. Results: During a median of 7.3 years of follow-up, 24,923 incident LC cases developed among 5,975,308 individuals. After adjusting for age, sex, smoking, alcohol, regular exercise, and body mass index, the adjusted hazard ratios (95% confidence intervals) for LC development were 1.39 (1.33–1.44) for the MetS-Developed group, 1.32 (1.26–1.37) for the MetS-Recovered group, and 1.51 (1.45–1.56) for the MetS-Sustained group, relative to the MetS-Free group. Stratified analyses according to age, sex, smoking, alcohol intake, exercise, diabetes mellitus, hypertension, dyslipidemia, and chronic kidney disease showed similar results. Conclusions: Both static and dynamic MetS status are independent risk factors for LC development. The risk of LC was the highest in people with sustained MetS and was lower in the MetS-Recovered group than in the MetS-Sustained group. These results suggest that improving a person’s MetS status may be helpful in preventing LC.

## 1. Introduction

Liver cirrhosis (LC) is the end-stage of various chronic liver diseases and is characterized by the pathological features of necro-inflammation and fibrosis with regenerative nodules [1]. It is a significant global health burden, triggering high morbidity and mortality rates; more than one million people die from LC [2,3], and patients with compensated cirrhosis and decompensated cirrhosis have a fivefold and tenfold increased risk of mortality, respectively, relative to the general population [4]. Aside from mortality, patients with LC experience a reduced quality of life and a high economic burden [5]. The etiologies of cirrhosis vary according to geographic region, with alcohol and nonalcoholic fatty liver disease (NAFLD) more common in Western and industrialized countries, while viral hepatitis is often found in China and other Asian countries [6]. 

Metabolic syndrome (MetS) is a cluster of metabolically related risk factors for cardiovascular disease [7]. Since MetS and NAFLD are known to be closely linked, sharing common pathophysiological mechanisms such as insulin resistance [8,9], NAFLD is considered to be the hepatic component of MetS [10]. As NAFLD progresses, hepatic inflammation, fibrosis, and cirrhosis can develop [11,12]. Recently, new terminology, “metabolic (dysfunction)-associated fatty liver disease,” has been introduced, and there has been an emphasis on the role of metabolic dysfunction in clinical outcomes of individuals with fatty liver disease [13,14,15]. Several studies have highlighted evidence that the components of MetS, including central obesity [16], dyslipidemia, and insulin resistance might be important factors for NAFLD [17]. Until now, only a few studies have investigated the associations between MetS and LC. One study that included biopsy-proven NAFLD patients showed that the presence of MetS was associated with a high risk of severe fibrosis with an odds ratio of 3.5, but this study was limited by its small number of participants [10]. Nderitu et al. reported that the components of MetS are associated with an increased risk of developing LC [18]. However, their study did not exclude subjects with a previous history of viral hepatitis, and outcome variables included both primary liver cancer and cirrhosis. Thus, we investigated the relationship between the characteristics of MetS status or a dynamic change in MetS components and the incidence of nonviral LC in a nationwide, population-based study. 

## 2. Materials and Methods

### 2.1. Data Source

The Korean government has a single mandatory health insurance system that covers nearly 97% of South Koreans. The Korean National Health Insurance Service (NHIS) manages all administrative processes and reimburses medical providers and pharmacies based on their claims data. The Korean NHIS is a mandatory social insurance that covers virtually all Koreans except for Medicaid beneficiaries in the lowest income bracket (who make up approximately 3% of the population). It conducts biennial health examinations for all Korean employees of any age and dependent subscribers who are older than 40 years also receive the examination every 2 years. It consists of anthropometric measurements, laboratory tests (i.e., lipid profiles, blood glucose measurement, liver function test), and questionnaires on lifestyle behaviors (e.g., smoking, alcohol consumption, and physical activity). Therefore, the Korean NHIS database includes health information from all Korean people based on eligibility, medical utilization, and results from health examinations. This database has been widely used for various epidemiologic studies [19,20].

### 2.2. Ethics Statement

The present study was approved by the institutional review board of the Seoul National University Hospital (no. E-2008-014-1145, 7 August 2020). Anonymized and de-identified information was used for analyses; therefore, the collection of informed consent was not required. The Korean NHIS database is open to use by all researchers whose study protocols are approved by the official review committee.

### 2.3. Study Population 

Among the 7,212,102 subjects (age ≥ 20 years) who participated in health screenings both from 2009 to 2010 and 2011 to 2012, respectively, we excluded individuals with missing data (*n* = 309,775) and those with pre-existing viral hepatitis (defined by the International Classification of Diseases, 10th revision (ICD-10), codes B15–B19), LC (defined by ICD-10 codes K703 and K746), or cancer diagnoses (*n* = 850,180). As patients who develop LC immediately after a health examination may present an unclear temporal relationship with MetS status as identified at the health examination, we allotted a one-year lag time and further excluded subjects diagnosed with LC or hepatitis within one year after their second health examination. Therefore, 5,975,308 subjects were included in the final study population. 

### 2.4. Exposure Variable: Metabolic Syndrome Status 

The definition of MetS was based on the 2009 agreement of the International Diabetes Federation and the American Heart Association/National Heart, Lung, and Blood Institute [7]. By definition, the presence of three or more out of the following five risk factors constituted a diagnosis of MetS: a triglyceride (TG) level of at least 150 mg/dL or the use of lipid-lowering medication; a high-density lipoprotein (HDL) cholesterol level of less than 40 mg/dL in men or less than 50 mg/dL in women or the use of lipid-lowering medication; a systolic blood pressure of at least 130 mmHg and/or diastolic blood pressure of at least 85 mmHg or the use of antihypertensive medication; a fasting glucose level of at least 100 mg/dL or the use of hypoglycemic agents; and abdominal obesity, which was defined as a waist circumference (WC) of at least 90 cm in men or at least 85 cm in women, according to the definition from the Korean Society for the Study of Obesity [21]. We divided participants into the following four groups using the change in MetS found between examinations performed from 2009 to 2010 and 2011 to 2012: MetS-Free, MetS-Developed, MetS-Recovery, and MetS-Sustained.

### 2.5. Outcome Variable 

As a study outcome variable, LC was defined using the ICD-10 codes K703 and K746, which cover alcoholic and other and unspecified cirrhosis, respectively. One or more diagnoses during hospitalization or two or more diagnoses in outpatient clinics were required for a cirrhosis diagnosis [22]. The study population was followed from the index date to the date of diagnosis of the study outcome, the censored date (e.g., outmigration), or 31 December 2018.

### 2.6. Covariates

As described previously [23], health behaviors, including smoking, alcohol consumption, and physical activity were evaluated by self-reporting questionnaires. Smoking history was classified as never, former, or current smoker. Alcohol consumption was divided into three levels: none, mild-to-moderate (<30 g of alcohol/day), and heavy (≥30 g/day). Regular physical activity was defined as moderate physical activity performed for more than 30 min daily and more than five days per week over the past week. Income status was divided into quartiles based on the amount of health insurance premiums paid (insurance premiums are determined by income level in South Korea), where those who received medical aid (the poorest 3% of the national population) were merged with the lowest income quartile. 

Body mass index (BMI) was calculated as weight in kilograms divided by height in meters squared. WC was measured at the midpoint between the lower margin of the ribs at the mid-axillary plane and the top of the iliac crest. Hypertension was defined as any of the following: a systolic blood pressure of at least 140 mmHg, a diastolic blood pressure of at least 90 mmHg, or treatment with an antihypertensive medication that was linked to the hypertension ICD-10 codes (I10–I13 and I15) and resulted in at least one claim in a year. Diabetes mellitus (DM) was defined as a blood glucose level of at least 126 mg/dL or a history of a hypoglycemic medication prescription that was linked to a diabetes ICD-10 code (E11–E14) and resulted in at least one claim in a year. Dyslipidemia was defined as a total cholesterol level of at least 240 mg/dL or a history of a lipid-lowering medication that was associated with the ICD-10 code E78. Chronic kidney disease (CKD) was defined as an estimated glomerular filtration rate of less than 60 mL/min/1.73 m² of the body surface area. Blood specimens including a liver function test such as aspartate transaminase (AST) and alanine transaminase (ALT) were obtained from each participant after an overnight fast lasting at least eight hours.

### 2.7. Statistical Analysis

The comparison of baseline characteristics according to the change in MetS was conducted using independent t-tests for continuous variables and the chi-squared test for categorical variables. The incidence rates of liver cirrhosis were assessed as incident cases divided by 1000 person-years. Cox proportional hazards regression was performed to estimate the risk of LC for each of the four MetS change groups (i.e., MetS-Free, MetS-Developed, MetS-Recovery, and MetS-Sustained). Multivariable analyses were adjusted for age, sex, smoking history, alcohol consumption, physical activity, and BMI. Stratified analyses were performed according to age (<65 vs. ≥65 years old), sex (male vs. female), smoking, alcohol (average drinking < 30 g/day vs. heavy drinking ≥ 30 g/day), and regular exercise (≥30 min of moderate physical activity at least five times per week or ≥20 min of strenuous physical activity at least three times per week). The statistical analyses were performed using SAS version 9.4 (SAS Institute Inc., Cary, NC, USA), and *p*-values of less than 0.05 were considered to be statistically significant.

## 3. Results

### 3.1. Characteristics of the Study Subjects

**Figure 1:**
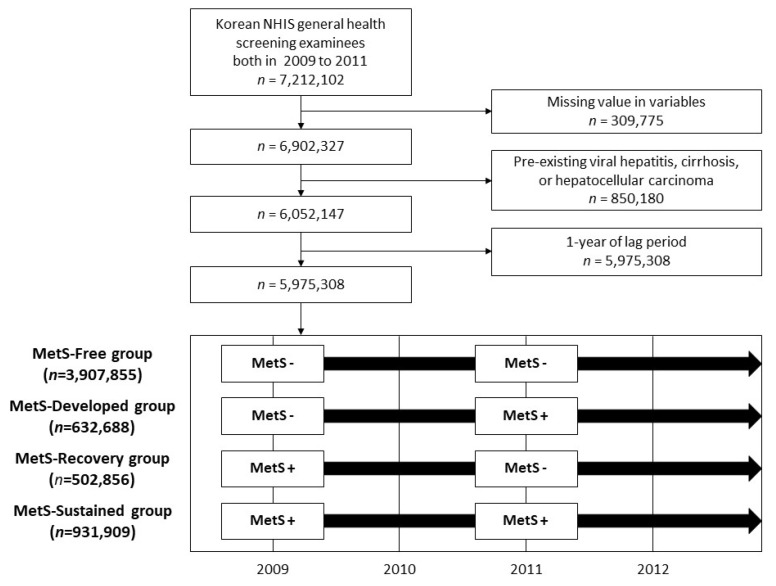
Flow chart of the study population selection process. MetS, metabolic syndrome.

A total of 5,975,308 subjects were enrolled in this study (Figure 1). In the total study participant group, 3,907,855 (65.4%) remained MetS-free during the first and the second national health examinations (MetS-Free group). Newly developed MetS at the time of the second screening was recorded in 632,688 (10.6%) (MetS-Developed group), and 502,856 (8.4%) participants had MetS at the first screening that normalized at the second screening (MetS-Recovery group). Sustained MetS across both screenings was noted in 931,909 (15.6%) (MetS-Sustained group).

The baseline characteristics among the four groups are shown in Table 1. The rate of heavy alcohol consumption was higher in the MetS-Developed group (9.7%), the MetS-Recovery group (9.6%), and the MetS-Sustained group (9.3%), than in the MetS-Free group (7.0%). The rate of regular physical activity was higher in the MetS-Developed group (20.1%) than in the other groups. The proportions of subjects with diabetes (27.8%), hypertension (62.3%), dyslipidemia (36.1%), and CKD (11.2%), respectively, were higher in the MetS-Sustained group than in the other groups. 

### 3.2. Incidence of LC According to Baseline MetS and Components

The median follow-up duration of the study population was 7.3 years. The incidence rate of LC for the normal group was 0.53 cases per 1000 person-years and 1.02 cases in the MetS group (Table 2). The risk of LC development was higher in the MetS group (adjusted hazard ratio (aHR): 1.36, 95% confidence interval (CI): 1.32–1.40). After adjustment for age, sex, smoking, alcohol, regular physical activity, and BMI, WC (aHR: 1.28, 95% CI: 1.23–1.32), fasting glucose (aHR: 1.50, 95% CI: 1.46–1.54), low HDL (aHR: 1.07, 95% CI: 1.04–1.10), and the presence of hypertension (aHR: 1.33, 95% CI: 1.29–1.37) were the significant risk factors for developing LC. Meanwhile, the baseline TG level was not significantly associated with LC development (aHR: 1.02, 95% CI: 0.99–1.04). When we performed a stratified analysis according to the baseline liver function test, an increased risk of LC was maintained in both groups; aHR: 1.15, 95% CI: 1.11–1.20 in AST < 40 and ALT < 40 and HR: 1.05, 95% CI: 1.01–1.10 in AST ≥ 40 or ALT ≥ 40 (Supplementary Table S1).

### 3.3. Incidence of LC According to the Change in MetS and Components

Table 3 shows the incidence of cirrhosis according to the change in MetS and its components. Relative to the MetS-Free group, the other groups showed a significantly higher risk of LC development. In particular, the risk of LC was increased significantly in the MetS-Developed group (aHR: 1.39, 95% CI: 1.33–1.44) as compared with the MetS-Free group. The risk of LC was also significantly increased among both the MetS-Recovery and MetS-Sustained groups, with the risk being higher in the MetS-Sustained group (aHR: 1.51, 95% CI: 1.45–1.56) than in the MetS-Recovery group (aHR: 1.32, 95% CI: 1.26–1.37).

Considering individual components, those who presented high values of WC (aHR: 1.13, 95% CI: 1.08–1.19), fasting glucose (aHR: 1.32, 95% CI: 1.27–1.37), and blood pressure (aHR: 1.27, 95% CI: 1.22–1.33), and low HDL (aHR: 1.13, 95% CI: 1.09–1.18) were at a significantly increased risk of LC development relative to those in the sustained normal group. Those who showed sustained high values of WC (aHR: 1.45, 95% CI: 1.39–1.51), fasting glucose (aHR: 1.71, 95% CI: 1.66–1.77), blood pressure (aHR: 1.46, 95% CI: 1.42–1.51), and TG (aHR: 1.05, 95% CI: 1.02–1.08) also demonstrated a greater risk of LC relative to those in the sustained normal group. Finally, those who showed normalization of WC (aHR: 1.14, 95% CI: 1.08–1.19), fasting glucose (aHR: 1.18, 95% CI: 1.32–1.23), and blood pressure (aHR: 1.22, 95% CI: 1.16–1.27) were at an elevated risk for LC relative to those in the sustained normal group, but the risk was lower than that in the sustained high group (Table 3).

### 3.4. Stratified Analyses

**Figure 2:**
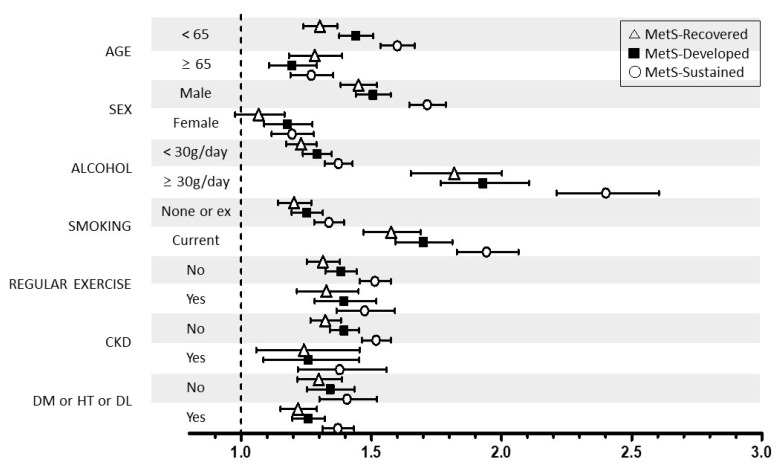
Relationship between the change in MetS status and liver cirrhosis stratified by patient characteristics. MetS, metabolic syndrome. CKD, chronic kidney disease; DM, diabetes mellitus; HT, hypertension; DL, dyslipidemia * Adjusted for age, sex, smoking, alcohol intake, and exercise.

We performed stratified analyses according to age (<65 vs. ≥65 years), sex (male vs. female), smoking, alcohol intake (<30 vs. ≥30 g/day), regular exercise, and the presence of DM, hypertension, dyslipidemia, and CKD. Stratified analyses also showed generally similar associations between the change in MetS and the risk of LC development (Figure 2). However, the association between sustained MetS or developed MetS and LC risk was more prominent in younger subjects (aHR: 1.60 and 1.44 vs. aHR: 1.27 and 1.20 in older subjects), male individuals (aHR: 1.71 and 1.51 vs. aHR: 1.20 and 1.18 in women), current smokers (aHR: 1.94 and 1.70 vs. aHR: 1.34 and 1.25 in nonsmokers), and heavy drinkers (aHR: 2.40 and 1.93 vs. aHR: 1.37 and 1.29 in non- or moderate drinkers).

## 4. Discussion

In this nationwide, population-based cohort study, we found that MetS status was an independent risk factor for LC development. We demonstrated that a baseline MetS status increased the LC risk by 36%, while a sustained MetS status further increased the risk of LC up to 51%. In addition, those who developed MetS showed a significantly greater risk of developing LC as compared to those who maintained a MetS-free status, suggesting the existence of a causal association between MetS and the development of LC. Among the components of MetS, central obesity, impaired fasting glucose, high blood pressure, and low HDL were associated with an increased risk of LC.

The most common primary etiologies for cirrhosis are known to be chronic hepatitis B, alcoholic liver disease, chronic hepatitis C, and NAFLD [24]. In recent years, with the rising incidence of obesity, NAFLD has become one of the leading causes of LC in some countries [25], and NAFLD-related LC may become the leading indication for liver transplantation [26]. Given the rising prevalence of MetS [27] and its association with NAFLD, it is likely that the prevalence of NAFLD-related LC will continue to rise. In addition, increased rates of hazardous alcohol consumption and the rising rates of obesity with its associated metabolic complications are likely to contribute to an ongoing growth in the number of patients with nonviral cirrhosis.

Although the mechanism regarding the association between MetS and the increase in LC has not been fully elucidated, glucose intolerance is thought to be at the center of the mechanism. Several studies have suggested an association between insulin resistance and hepatic fibrosis. Chronic hyperinsulinemia, induced by insulin resistance, directly activates stellate cells [28,29], Moreover, this hyperinsulinemia disrupts insulin signaling and induces lipid accumulation in the skeletal muscles and liver [30]. Insulin resistance–induced hepatic lipid accumulation and reactive oxygen species indirectly activate stellate cells and initiate cellular-signaling cascades, triggering hepatic fibrosis [31]. Furthermore, insulin resistance causes adipose tissue to produce excess free fatty acids, accelerating lipid accumulation in the liver, which leads to lipotoxicity, which in turn can trigger a vicious cycle that exacerbates insulin resistance [32]. Although we could not evaluate the fatty liver status in this study, fatty-liver-disease-related liver inflammation and the subsequent post-inflammatory processes may contribute to the development of LC. In a European cohort study, patients with nonalcoholic steatohepatitis were at a significantly higher risk of acquiring LC compared to controls with a HR of 22.67 and those with NAFLD showed a HR of 5.83 [33]. Since MetS and fatty liver disease are closely linked, further studies are needed to determine the mechanism regarding the association between MetS and LC, in terms of fatty liver.

In this study, when the risk of LC was analyzed by changes in individual MetS components, the development of central obesity, impaired fasting glucose, low HDL, and high blood pressure led to a significantly increased risk of LC development, with impaired fasting glucose among them correlated with the largest increase in LC risk. This shows that glucose intolerance plays a key role in the influence of MetS on LC. By individual components, a raised TG level, low total cholesterol, a low low-density lipoprotein level, raised glucose, diabetes, and a low HDL level were also associated with an increased risk of cirrhosis in a Swedish cohort study [18]. Consistently, high glucose and low HDL levels were associated with a greater risk of LC; however, there was no statistical significance in the association between a high TG level and LC in this study. Previously, Chen et al. reported that hyper-TG reduces the risk for advanced fibrosis [34], suggesting that a decline in TG production exists in patients with advanced liver fibrosis. Thus, more clinical and experimental studies are needed regarding the relationship between the TG level and LC.

To the best of our knowledge, this is the first study to assess the risk of LC according to MetS state changes. Recovery from MetS was associated with a reduced risk of developing LC as compared with sustained MetS, suggesting that improving MetS status may have a preventive effect against LC development. On the other hand, the risk of LC was higher in the MetS-Recovery group than in the MetS-Free group, although both groups were equally free of MetS during the follow-up health examination period, indicating that a history of MetS, as well as current MetS status, influences LC development.

The association between sustained or developed MetS and LC risk was more prominent in men, those aged younger than 65 years, and those who were heavy drinkers and/or current smokers, suggesting a greater focus is necessary on these high-risk individuals. As the MetS status can be dynamically changed [35], a comprehensive evaluation of the dynamic status of MetS is required to stratify the risk of LC. Therefore, this study may be helpful for defining appropriate strategies among individuals with MetS regarding risk stratification and early detection of LC.

There were several limitations to this study. First, as is true with all epidemiological studies, it is difficult to ascertain causality. Second, although we used the Korean NHIS database, which includes the entire Korean population, and a large number of study participants underwent national health examinations (66.0% in 2009) [36], there may be a selection bias in that the analysis was performed only for individuals who actually underwent the national health examination. Third, although cirrhosis is a clinically and pathologically defined disease, the diagnosis of LC was identified based on ICD codes in this study; therefore, misdiagnosis or omission may be possible. Our definition of LC reflects clinically significant LC because patients with early LC are often asymptomatic and have nonspecific symptoms. In Korea, LC is either often diagnosed during the workup process for jaundice, abdominal distension, or an abnormality in a liver function test [37], or during a regular surveillance program provided free of charge in patients with chronic viral hepatitis [38]. Easy access to healthcare through universal health coverage, availability of a national health examination, which includes AST/ALT and GGT [39], and a national liver cancer surveillance program might increase the chances of detecting clinically significant LC. In addition, the definition of LC according to ICD 10 codes has been accepted as sufficiently reliable and has been used in previous studies [22,40], although external validation with clinical data has not been conducted. Finally, there may be a recall bias and impreciseness regarding the self-reported questionnaire. However, it is difficult to apply objective exams to large-scale retrospective studies, and self-reporting is the only practical method. As in most preceding studies based on self-reports [41,42,43], the best method available in this study was using a self-report questionnaire, and despite the impreciseness of self-report method, the results are reasonably reliable.

In conclusion, the dynamic status of MetS is closely associated with the risk of nonviral LC, and the risk was greater when MetS was sustained. Recovered MetS lowers the risk of developing LC as compared with sustained MetS, and the history of MetS, as well as the current status of MetS, influences the risk of LC. Since dynamic changes in MetS affect the risk of LC, a comprehensive evaluation of the dynamic status of MetS is required and careful attention should be paid to the high-risk group.

## Figures and Tables

**Table 1:** Baseline characteristics of the study population.

	Change in the Presence of Metabolic Syndrome across 2 Years				
	MetS-Free	MetS-Developed	MetS-Recovery	MetS-Sustained	*p*-Value
Number of subjects	3,907,855	632,688	502,856	931,909	
Age, years	43.35 ± 12.73	50.21 ± 13.02	50.83 ± 12.9	55.12 ± 12.54	<0.0001
Male (%)	2,229,163 (57.0)	389,660 (61.6)	311,410 (61.9)	500,751 (53.7)	<0.0001
Smoking status					<0.0001
Never-smoker (%)	2,301,854 (58.9)	342,536 (54.1)	274,620 (54.6)	555,648 (59.6)	
Ex-smoker (%)	566,236 (14.5)	103,611 (16.4)	90,146 (17.9)	152,539 (16.4)	
Current smoker (%)	1,039,765 (26.6)	186,541 (29.5)	138,090 (27.5)	223,722 (24.0)	
Alcohol consumption					<0.0001
None (%)	1,869,729 (47.9)	313,789 (49.6)	253,826 (50.5)	529,023 (56.8)	
Mild-to-moderate (%)	1,766,562 (45.2)	257,585 (40.7)	200,679 (39.9)	315,903 (33.9)	
Heavy (%)	271,564 (7.0)	61,314 (9.7)	48,351 (9.6)	86,983 (9.3)	
Regular physical activity (%)	711,761 (18.2)	127,323 (20.1)	94,936 (18.9)	182,300 (19.6)	<0.0001
Body weight, kg	61.99 ± 10.4	67.41 ± 11.59	68.19 ± 11.76	69.98 ± 12.69	<0.0001
BMI, kg/m^2^	22.71 ± 2.72	24.88 ± 2.84	25.18 ± 4.47	26.35 ± 4.32	<0.0001
WC, cm	77.26 ± 8.12	83.15 ± 7.67	85.33 ± 8.64	87.98 ± 8.49	<0.0001
SBP, mmHg	118.53 ± 13.24	125.03 ± 14.14	130.19 ± 13.64	132.07 ± 14.8	<0.0001
DBP, mmHg	74.25 ± 9.19	78.14 ± 9.52	80.87 ± 9.56	81.57 ± 10.11	<0.0001
Comorbidities					
Hypertension (%)	419,958 (10.8)	187,784 (29.7)	194,318 (38.6)	580,159 (62.3)	<0.0001
Diabetes (%)	94,710 (2.4)	49,985 (7.9)	61,823 (12.3)	259,137 (27.8)	<0.0001
Dyslipidemia (%)	309,074 (7.9)	107,062 (16.9)	118,634 (23.6)	336,659 (36.1)	<0.0001
Chronic kidney disease (%)	212,219 (5.4)	46,121 (7.3)	41,580 (8.3)	104,651 (11.2)	<0.0001
Laboratory results					
Fasting glucose (mg/dL)	91.56 ± 15.01	97.22 ± 22.53	105.09 ± 24.88	112.45 ± 33.7	<0.0001
Total cholesterol (mg/dL)	190.65 ± 36.63	204.78 ± 41.07	204.54 ± 46.89	206.32 ± 47.62	<0.0001
Triglycerides (mg/dL)	92 (66–128)	125 (94–169)	173 (128–227)	182 (131–253)	<0.0001
HDL-cholesterol (mg/dL)	58.99 ± 32.12	55.2 ± 33.62	49.89 ± 31.84	49.48 ± 32.44	<0.0001
Creatinine (mg/dL)	1.16 ± 1.51	1.14 ± 1.42	1.17 ± 1.54	1.12 ± 1.4	<0.0001
AST (IU/L)	21 (18–26)	24 (20–29)	24 (20–30)	25 (21–31)	<0.0001
ALT (IU/L)	18 (14–25)	22 (17–32)	24 (17–35)	25 (18–37)	<0.0001

Abbreviations: MetS, metabolic syndrome; LC, liver cirrhosis; BMI, body mass index; WC, waist circumference; SBP, systolic blood pressure; DBP, diastolic blood pressure; HDL, high-density lipoprotein. Values are presented as mean ± standard deviation or median (range) for continuous variables and number (%) for categorical variables.

**Table 2:** Incidence of liver cirrhosis according to metabolic syndrome and its components.

	No. of Subjects	LC Cases (*n*)	Incidence of LC (1000 Person-Years)	Crude HR (95% CI)	Adjusted HR Model 1 ^b^ (95% CI)	Adjusted HR Model 2 ^c^ (95% CI)
Metabolic syndrome ^a^						
No	4,410,711	14,901	0.555	1 (reference)	1 (reference)	1 (reference)
Yes	1,564,597	10,022	1.017	1.91 (1.86–1.96)	1.43 (1.39–1.46)	1.36 (1.32–1.40)
MetS components						
Waist circumference						
No	4,368,462	16,419	0.594	1 (reference)	1 (reference)	1 (reference)
Yes	1,606,846	8504	0.837	1.41 (1.37–1.44)	1.31 (1.27–1.34)	1.50 (1.46–1.54)
Fasting glucose						
No	4,078,226	12,519	0.484	1 (reference)	1 (reference)	1 (reference)
Yes	1,897,082	12,404	1.040	2.15 (2.10–2.21)	1.57 (1.53–1.61)	1.50 (1.46–1.54)
HDL cholesterol						
No	4,514,420	18,105	0.634	1 (reference)	1 (reference)	1 (reference)
Yes	1,460,888	6818	0.738	1.16 (1.13–1.19)	1.41 (1.37–1.45)	1.33 (1.29–1.37)
Blood pressure						
No	3,256,666	8999	0.435	1 (reference)	1 (reference)	1 (reference)
Yes	2,718,642	15,924	0.901	2.14 (2.08–2.19)	1.41 (1.37–1.45)	1.33 (1.29–1.37)
Triglycerides						
No	3,905,281	14,455	0.585	1 (reference)	1 (reference)	1 (reference)
Yes	2,070,027	10,468	0.800	1.37 (1.33–1.40)	1.12 (1.09–1.15)	1.02 (0.99–1.04)

Abbreviations: LC, liver cirrhosis; HDL, high-density lipoprotein; HR, hazard ratio; CI, confidence interval; BMI, body mass index; MetS, metabolic syndrome. ^a^ Metabolic syndrome and components were defined from blood tests and anthropometric measurements from the 2009−2010 examinations as follows: waist circumference ≥ 90 cm (male) or 85 cm (female), systolic blood pressure ≥ 130 mmHg and/or diastolic blood pressure ≥ 85 mmHg, fasting glucose ≥ 100 mg/dL, triglycerides ≥ 150 mg/dL, and HDL < 40 mg/dL (male) or 50 mg/dL (female). The presence of three or more out of these five components was regarded as definitive for MetS. ^b^ Model 1: adjusted for age and sex. ^c^ Model 2: adjusted age, sex, smoking, alcohol, regular physical activity, and BMI.

**Table 3:** Incidence of liver cirrhosis according to metabolic change during two years of follow-up.

	Number of Subjects	Number of LC Cases	Incidence of LC (1000 Person-Years)	Crude HR (95% CI)	Adjusted HR Model 1 ^b^ (95% CI)	Adjusted HR Model 2 ^c^ (95% CI)
Metabolic syndrome ^a^ status						
MetS-Free	3,907,855	12,117	0.459	1 (reference)	1 (reference)	1 (reference)
MetS-Developed	632,688	3615	0.905	1.85 (1.78–1.92)	1.43 (1.38–1.48)	1.39 (1.33–1.44)
MetS-Recovery	502,856	2784	0.878	1.79 (1.72–1.87)	1.35 (1.30–1.41)	1.32 (1.26–1.37)
MetS- Sustained	931,909	6407	1.093	2.23 (2.17–2.30)	1.56 (1.51–1.61)	1.51 (1.45–1.56)
Changes in MetS components						
Waist circumference						
No → No	3,923,615	14,381	0.579	1 (reference)	1 (reference)	1 (reference)
No → Yes	513,925	2172	0.668	1.15 (1.10–1.21)	1.12 (1.07–1.17)	1.13 (1.08–1.19)
Yes → No	444,847	2038	0.726	1.25 (1.20–1.31)	1.12 (1.07–1.17)	1.14 (1.08–1.19)
Yes → Yes	1,092,921	6332	0.916	1.58 (1.53–1.63)	1.42(1.38-1.47)	1.45 (1.39–1.51)
Fasting glucose						
No → No	3,359,245	9497	0.445	1 (reference)	1 (reference)	1 (reference)
No → Yes	798,529	3866	0.767	1.72 (1.66–1.79)	1.39 (1.34–1.44)	1.32 (1.27–1.37)
Yes → No	718,981	3022	0.665	1.49 (1.43–1.56)	1.22 (1.17–1.27)	1.18 (1.13–1.23)
Yes → Yes	1,098,553	8538	1.241	2.79 (2.71–2.87)	1.82 (1.76–1.87)	1.71 (1.66–1.77)
HDL cholesterol						
No → No	3,906,588	15,471	0.626	1 (reference)	1 (reference)	1 (reference)
No → Yes	672,699	3302	0.776	1.24 (1.19–1.29)	1.12 (1.07–1.16)	1.13 (1.09–1.18)
Yes → No	607,832	2634	0.685	1.09 (1.05–1.14)	1.05 (1.01–1.10)	1.06 (1.02–1.11)
Yes → Yes	788,189	3516	0.705	1.12 (1.08–1.17)	1.02 (0.98–1.06)	1.04 (1.00–1.08)
Blood pressure						
No → No	2,599,455	6367	0.385	1 (reference)	1 (reference)	1 (reference)
No → Yes	774,397	3340	0.682	1.77 (1.70–1.84)	1.35 (1.29–1.41)	1.27 (1.22–1.33)
Yes → No	657,211	2632	0.633	1.64 (1.57–1.72)	1.27 (1.21–1.33)	1.22 (1.16–1.27)
Yes → Yes	1,944,245	12,584	1.030	2.67 (2.59–2.76)	1.57 (1.52–1.62)	1.46 (1.42–1.51)
Triglycerides						
No → No	3,241,284	11,201	0.546	1 (reference)	1 (reference)	1 (reference)
No → Yes	773,869	3477	0.710	1.30 (1.25–1.35)	1.08 (1.04–1.12)	1.00 (0.97–1.04)
Yes → No	663,997	3254	0.777	1.42 (1.37–1.48)	1.13 (1.08–1.17)	1.06 (1.02–1.10)
Yes → Yes	1,296,158	6991	0.854	1.57 (1.52–1.61)	1.19 (1.15–1.22)	1.05 (1.02–1.08)

Abbreviations: LC, liver cirrhosis; HDL, high-density lipoprotein; HR, hazard ratio; CI, confidence interval; BMI, body mass index; MetS, metabolic syndrome. ^a^ Metabolic syndrome and components were defined from blood tests and anthropometric measurements from the 2009−2010 examinations as follows: waist circumference ≥ 90 cm (male) or 85 cm (female), systolic blood pressure ≥ 130 mmHg and/or diastolic blood pressure ≥ 85 mmHg, fasting glucose ≥ 100 mg/dL, triglycerides ≥ 150 mg/dL, and HDL < 40 mg/dL (male) or 50 mg/dL (female). The presence of three or more out of these five components was regarded as definitive for MetS. ^b^ Model 1: adjusted for age and sex. ^c^ Model 2: adjusted age, sex, smoking, alcohol, regular physical activity, and BMI.

## Data Availability

Restrictions apply to the availability of all data analyzed during this study because they were used under license. The corresponding author will on request detail the restrictions and any conditions under which access to some data may be provided. (https://nhiss.nhis.or.kr/REQ000042255-001, accessed on 4 November 2021).

## Supplementary Materials

The following are available online at https://www.mdpi.com/article/10.3390/biomedicines9121948/s1, Table S1: Risk of liver cirrhosis according to metabolic syndrome and its components stratified by baseline liver function.

## Abbreviations

MetS: metabolic syndrome; LC, liver cirrhosis; NAFLD, nonalcoholic fatty liver disease; NHIS, National Health Insurance Service; ICD-10, International Classification of Diseases, 10th revision; DM, diabetes mellitus; CKD, chronic kidney disease; aHR, adjusted hazard ratio; CI: confidence interval; BMI, body mass index; TG, triglyceride; HDL, high-density lipoprotein; WC, waist circumference.
