# Supplementary material for: The Change in Metabolic Syndrome Status and the Risk of Nonviral Liver Cirrhosis
Source: Biomedicines. 2021 Dec 20;9(12):1948. doi: 10.3390/biomedicines9121948 (PMC8698513; doi:10.3390/biomedicines9121948)
Supplement: Supplementary file 1 [file biomedicines-09-01948-s001.zip › biomedicines-1471778-supplementary.pdf]

Supplementary Table S1. Risk of liver cirrhosis according to metabolic syndrome and its components stratified by baseline liver function

	AST <40 and ALT<40			AST ≥40 or ALT ≥40		
	LC cases (n)	Incidence of LC (1000 person-years)	Adjusted HR (95% CI)	LC cases (n)	Incidence of LC (1000 person-years)	Adjusted HR (95% CI)
**Metabolic syndrome**[a]						
No	9055	0.358	1 (reference)	5846	2.222	1 (reference)
Yes	4901	0.632	1.15 (1.11–1.20)	5121	2.427	1.05 (1.01–1.10)
**MetS components**						
Waist circumference						
No	9382	0.378	1 (reference)	7037	2.516	1 (reference)
Yes	4496	0.552	1.19 (1.14–1.25)	4008	1.974	1.14 (1.09–1.20)
Fasting glucose						
No	7809	0.337	1 (reference)	4710	1.739	1 (reference)
Yes	6069	0.619	1.28 (1.23–1.32)	6335	2.989	1.39 (1.34–1.44)
HDL cholesterol						
No	10,130	0.402	1 (reference)	7975	2.374	1 (reference)
Yes	3759	0.481	1.03 (1.00–1.07)	3,059	2.152	0.91 (0.87–0.95)
Blood pressure						
No	5569	0.298	1 (reference)	3,430	1.715	1 (reference)
Yes	8310	0.581	1.18 (1.13–1.22)	7,614	2.693	1.22 (1.17–1.28)
Triglycerides						
No	8845	0.389	1 (reference)	5,610	2.866	1 (reference)
Yes	5142	0.497	0.94 (0.91–0.98)	5,326	1.941	0.71 (0.68–0.74)

Abbreviations: LC, liver cirrhosis; HDL, high-density lipoprotein; HR, hazard ratio; CI, confidence interval; BMI, body mass index; MetS, metabolic syndrome; AST, aspartate aminotransferase; ALT, alanine aminotransferase. Metabolic syndrome and components were defined from blood tests and anthropometric measurements from the 2009−2010 examinations as follows: waist circumference ≥ 90 cm (male) or 85 cm (female), systolic blood pressure ≥ 130 mmHg and/or diastolic blood pressure ≥ 85 mmHg, fasting glucose ≥ 100 mg/dL, triglycerides ≥ 150 mg/dL, and HDL < 40 mg/dL (male) or 50 mg/dL (female). The presence of three or more out of these five components was regarded as definitive for MetS. Adjusted age, sex, smoking, alcohol, regular physical activity, and BMI.
